# High Membranous Expression of Fatty Acid Transport Protein 4 Is Associated with Tumorigenesis and Tumor Progression in Clear Cell Renal Cell Carcinoma

**DOI:** 10.1155/2019/5702026

**Published:** 2019-04-04

**Authors:** Young-Sik Kim, Jiyoon Jung, Hoiseon Jeong, Ju-Han Lee, Hwa Eun Oh, Eung Seok Lee, Jung-Woo Choi

**Affiliations:** Department of Pathology, Korea University Ansan Hospital, Ansan, Republic of Korea

## Abstract

The increased requirement of fatty acids forces cancer cells to enhance uptake of fatty acids from the extracellular milieu, in addition to *de novo* lipogenesis. Coexpression of cluster of differentiation 36 (CD36) with fatty acid transport protein 4 (FATP4) or long-chain acyl CoA synthetase 1 (ACSL1) synergistically activated fatty acid uptake in experimental models. In this study, we investigated the immunohistochemical expression of CD36, FATP4, and ACSL1 in 180 cases of clear cell renal cell carcinoma (RCC) in comparison with 80 specimens of the normal kidney. We also examined the clinical implication of these three fatty acid transporters in RCC, which was validated by an open-access The Cancer Genome Atlas data analysis. Both CD36 and FATP4 revealed higher membranous expressions in RCC tumor cells than in normal cells. In contrast, ACSL1 expression was remarkably reduced in RCC tumor cells compared to normal cells. CD36, FATP4, and ACSL1 showed high expressions in 74 (41.1%), 85 (47.2%), and 72 (40.0%) out of 180 RCC cases, respectively. Clinically, high FATP4 in tumor cells was associated with female gender (*p* = 0.05), high TNM stage (*p* = 0.039), tumor necrosis (*p* = 0.009), and tumor recurrence (*p* = 0.037), while high ACSL1 was only related to female gender (*p* = 0.023). CD36 expression revealed no correlation with the clinicopathologic parameters of RCC. Increased FATP4 expression displayed an association with short recurrence-free survival (*p* = 0.003). In conclusion, the high FATP4 expression was clinically associated with poor prognostic factors of RCC. Overexpression of membranous FATP4 and CD36 combined with reduced cytoplasmic expression of ACSL1 might be a tumor-specific feature of RCC, contributing to the tumorigenesis and tumor progression.

## 1. Introduction

Rapidly proliferating cancer cells have high demand for energy and macromolecules, and they accordingly reprogram their cellular metabolism to consume nutrients such as glucose, glutamine, and fatty acids more efficiently [1]. Specifically, fatty acids support tumorigenesis by supplying signaling molecules, basic elements for lipid biosynthesis, and sources of metabolic energy such as adenosine triphosphate [2, 3]. The increased requirement for fatty acids forces cancer cells to upregulate *de novo* lipogenesis, lipolysis of intracellular lipid droplets, or uptake of fatty acids from the extracellular milieu, depending on the cell type or specific microenvironment.

Clear cell renal cell carcinoma (RCC) is the most common type of renal malignancy and is characterized histologically by a clear cytoplasm containing lipids and glycogen [4]. Increased cytoplasmic lipid content could imply modified lipid metabolism in RCC tumor cells, unlikely in normal cells. A recent study has reported that only 15% of RCC cases showed increased expression of fatty acid synthase, which is mandatory for lipogenesis [5]. Another study showed that hypoxia-inducible factor- (HIF-) 1*α*, which is not degraded in RCC, enhanced the expression of a very low-density lipoprotein receptor, leading to uptake and accumulation of intracellular lipids [6]. Thus, some RCC tumor cells might metabolically depend on other mechanisms to provide fatty acids, such as uptake from extracellular sources, although the significance of intracellular lipids has not been clearly elucidated.

For the mechanism of cellular fatty acid uptake, passive diffusion, protein-facilitated transport, and/or vectorial acylation have been demonstrated. The most characterized protein that facilitates fatty acid uptake is cluster of differentiation 36/fatty acid translocase (CD36/FAT) [7]. High membranous expression of CD36 attracts fatty acids close to the outer plasma membrane and lowers the activation energy required for fatty acids to cross the lipid bilayer, thereby promoting cellular fatty acid uptake. Clinically, CD36 expression in tumor cells has been proposed to induce tumor progression in various human cancers [8]. Multiple studies have revealed that CD36-positive cancer cells initiated metastasis via heightened lipid metabolism [9]. Fatty acid transport protein 4 (FATP4) and long-chain acyl CoA synthetase 1 (ACSL1) belong to a mammalian family of long-chain fatty acyl CoA synthetases, which activate fatty acids by esterification with coenzyme A [10]. Unlike ACSL1, FATP4 is much more proficient in activating very-long-chain fatty acids and carries a transporter function in itself. While ACSL1 mRNA was frequently reported to be downregulated in multiple human malignancies, the oncogenic or tumor-suppressive function of ACSL1 differed considerably across cancer types [11]. Regarding FATP4, there has been a functional study showing that the overexpression of acyl CoA synthetases such as FATP2, FATP4, and ACSL1 as well as CD36 enhanced fatty acid uptake in human hepatoma cells [12]. In addition, Yen et al. described that the high FATP4 expression was associated with tumor progression in human breast cancer through epithelial-mesenchymal transition [13]. Recently, it was reported that the coexpression of CD36 with FATP4 or ACSL1 synergistically activated fatty acid uptake because CD36 directly facilitates fatty acid transport across the plasma membrane, and FATP4 or ACSL1 traps the transported intracellular fatty acid in the form of acyl-CoA [14].

In this study, we evaluated the differential immunohistochemical expression of fatty acid transporters including CD36, FATP4, and ACSL1 between RCC tumor cells and normal renal tubular epithelial cells. Furthermore, we examined the clinicopathologic implication of CD36, FATP4, and ACSL1 expressions in RCC cases. Lastly, we analyzed overall survival (OS) of RCC patients in terms of CD36, FATP4, and ACSL1 expressions by an open-access The Cancer Genome Atlas (TCGA) data analysis with the help of the OncoLnc tool.

## 2. Materials and Methods

### 2.1. Tissue Samples

One hundred and eighty patients with RCC consecutively resected with partial or radical nephrectomy from 1996 to 2013 at the Department of Pathology, Korea University Ansan Hospital, were registered in the study. In addition, 80 samples of normal kidney tissues surgically resected for nonneoplastic or neoplastic diseases were collected for comparison. In all cases, representative tissue samples were fixed in 10% buffered formalin, embedded in paraffin, cut, and stained with hematoxylin and eosin (H&E) for histological evaluation. After reviewing each case, the most representative section was selected by two pathologists (JW Choi and YS Kim). Tumor stage was classified as low (I and II) or high (III and IV) according to the tumor-node-metastasis (TNM) classification system of the American Joint Committee on Cancer [15]. Based on the criteria by Fuhrman et al., the nuclear grading was divided into low (1 and 2) and high (3 and 4) [16]. Other clinical information such as metastasis, recurrence, and cause of death was available in all cases.

### 2.2. Tissue Microarray Construction

When reviewing the H&E-stained sections from each formalin-fixed, paraffin-embedded block, we searched for the most diagnostic areas. Three representative 1 mm cores obtained from each case were inserted in a grid pattern into a recipient paraffin block using a tissue arrayer (Beecher Instruments Inc., Sun Prairie, WI, USA).

### 2.3. Immunohistochemistry

Immunohistochemistry was performed using an automated immunostainer (Bond-Max™ Leica Microsystems, Melbourne, Australia), as described by the manufacturer's protocol. Representative tissue sections were deparaffinized and dehydrated, and heat pretreatment was performed for 20 min. After incubation with peroxide for 10 min, the slides were stained with antibodies against CD36 (clone D8L9T, Cell Signaling Technology, Danvers, MA, USA), FATP4 (clone EPR17319, Abcam, Cambridge, UK), and ACSL1 (clone EPR13499, Abcam, Cambridge, UK). The working dilution of each antibody was 1 : 400, 1 : 100, and 1 : 200, respectively. After incubation with secondary antibodies at room temperature for 10 min, the sections were developed with 3,3′-diaminobenzidine and counterstained with Harris hematoxylin.

### 2.4. Image Database Construction

Digital images were acquired on an Olympus BX53 light microscope using a DP70 color digital camera with 12-megapixel resolution, which was operated with the cellSens™ Standard Microsoft imaging software (Olympus Corporation, Tokyo, Japan). Three microscopic images from each tissue microarray core were taken at 40x, and they were saved in JPEG format at a pixel resolution of 1360 × 1024.

### 2.5. Quantitative Scoring

For CD36 and FATP4, only cell membrane staining was regarded as significant. The measurement was performed by the ImmunoMembrane (IM) program, which is a publicly available web application for digital analysis of membrane staining. A quantitative score (IM score, 0–20 points) was generated by the sum of the membrane completeness (0–10 points) and the membrane intensity (0–10 points) [17]. Cases with IM scores of higher than 15 and 12 were considered as a high expression of CD36 and FATP4, respectively. For ACSL1, as it shows only cytoplasmic staining, the percentage of the positively stained area to the total image area was calculated by ImageJ 1.49v (Wayne Rasband, National Institutes of Health, USA). Cases with a percentage of greater than 0.1 were recorded as a high expression of ASCL1. Common to all of these proteins, each cutoff value was determined by receiver operating characteristic curves, which calculate the sensitivity and specificity based on the TNM stage. For each case, the tissue microarray core with the highest score was selected for statistical analysis.

### 2.6. TCGA Analysis Using the OncoLnc

The OncoLnc (http://www.oncolnc.org/) is an online tool developed for exploring OS of various cancer patients downloaded from TCGA data according to mRNA, miRNA, and lncRNA expression [18]. This tool enables researchers to generate Kaplan-Meier plots stratified by gene expression levels and corresponding log-rank *p* values. The RCC patient's cohort (KIRC) stored in the OncoLnc database included 522 cases. The gene symbols of *CD36*, *SLC27A4*, and *ACSL1* were entered to represent CD36, FATP4, and ACSL1, respectively. Upper percentiles of 40, 30, and 40 were selected as the cutoff value dividing the high and low expressions of CD36, FATP4, and ACSL1, respectively.

### 2.7. Statistical Analysis

The protein expressions of CD36, FATP4, and ACSL1 were compared for patient characteristics using the chi-square test or Fisher's exact test. The differential expression of CD36, FATP4, and ACSL1 between normal renal tubular epithelial cells and RCC tumor cells was determined with Student's *t*-test. Overall and recurrence-free survivals were analyzed with Kaplan-Meier curves and tested with the log-rank test. The influence of possible confounding factors was analyzed by a Cox proportional hazards model using a nonstepwise method. Statistical significance was defined as *p* < 0.05. All statistical analyses were performed with SPSS for Windows 10.0 (SPSS Inc., Chicago, IL, USA).

## 3. Results

### 3.1. Patients

The study population consisted of 127 (70.6%) men and 53 (29.4%) women with a median age at diagnosis of 58 years (range: 25 to 83 years). Based on Fuhrman nuclear grading, 34 (18.9%) patients corresponded to low (2, grade 1; 32, grade 2) and 146 (81.1%) patients to high (113, grade 3; 33, grade 4). 145 (80.6%) patients were classified as low stage (117, stage I; 28, stage II), while 35 (19.4%) patients belonged to high stage (25, stage III; 10, stage IV). Sarcomatoid differentiation and tumor necrosis were detected in 9 (5.0%) and 20 (11.1%) cases, respectively, and 10 (5.6%) patients had distant metastasis at diagnosis. In addition, 33 (18.3%) patients experienced tumor recurrence, which was defined as local recurrence or distant metastasis evaluated by clinical, radiological, or histological confirmation. The median duration of follow-up was 40.7 months (range: 1 to 173 months).

### 3.2. CD36, FATP4, and ACSL1 Expression in Normal and Tumor Tissues

**Figure 1:**
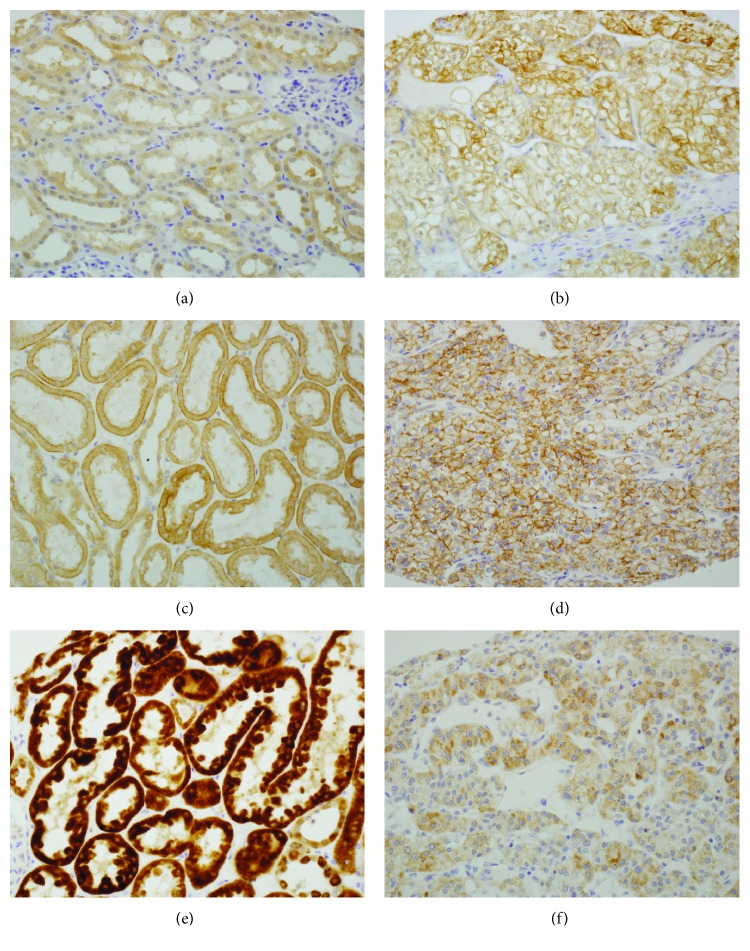
CD36 was weakly positive in the cytoplasm of tubular epithelial cells (a), while tumor cells show strong membranous expression (b). FATP4 was weakly positive in the cytoplasm and cell membrane of renal tubules, relatively prominent at the basal and apical membranes (c). In RCC tumor cells, FATP4 revealed higher membranous expression than that in normal cells (d). ACSL1 showed diffuse cytoplasmic staining in renal tubules (e) and RCC tumor cells (f), while the staining was remarkably reduced in tumor tissues.

Normal renal tubular epithelial cells were weakly positive for CD36 and FATP4 in their cytoplasm and/or cell membrane (Figures 1(a) and 1(c)). CD36 was also strongly positive in the cytoplasm of adjacent vascular endothelial cells. Typically, membranous expression of FATP4 in normal tubular cells was accentuated at the basal and apical membranes rather than at the basolateral membrane. In RCC tumor cells, both CD36 and FATP4 revealed predominant membranous expression, which was significantly higher than in normal cells (Table 1), and showed loss of the site-specific localization pattern found in normal cells (Figures 1(b) and 1(d)). For ACSL1, normal and RCC tumor cells showed diffuse cytoplasmic staining only (Figures 1(e) and 1(f)). However, unlike that of CD36 and FATP4, the staining intensity of ACSL1 was remarkably reduced in tumor tissues compared to normal cells.

### 3.3. Clinicopathologic Implication of CD36, FATP4, and ACSL1 Expression in RCC

CD36, FATP4, and ACSL1 showed a high expression in 74 (41.1%), 85 (47.2%), and 72 (40.0%) out of 180 RCC cases, respectively (Table 2). Clinically, high FATP4 in tumor cells was associated with female gender (*p* = 0.05), high TNM stage (*p* = 0.039), tumor necrosis (*p* = 0.009), and tumor recurrence (*p* = 0.037), while high ACSL was only related to female gender (*p* = 0.023). CD36 expression revealed no correlation with the clinicopathologic parameters of RCC.

**Figure 2:**
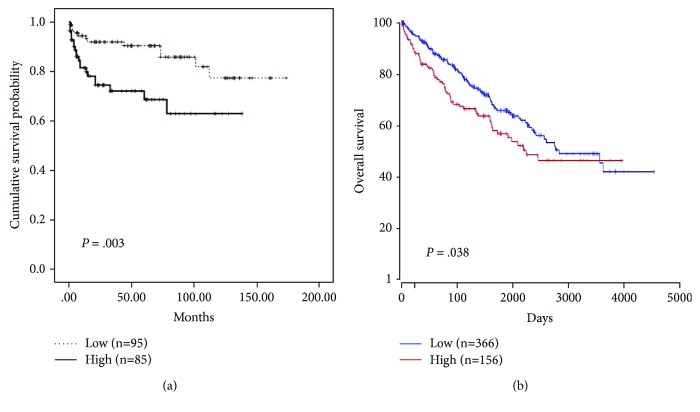
High FATP4 expression displayed a significant association with short recurrence-free survival in RCC cases from this study cohort (a). The Cancer Genome Atlas data analysis by the OncoLnc tool showed that the high FATP4 expression was correlated with worse overall survival in RCC patients (b).

In univariate analyses, high TNM stage (*p* = 0.035), tumor necrosis (*p* < 0.001), sarcomatoid feature (*p* = 0.002), and distant metastasis (*p* < 0.001) were associated with poor OS. High grade (*p* = 0.014), high TNM stage (*p* < 0.001), distant metastasis (*p* < 0.001), tumor necrosis (*p* < 0.001), sarcomatoid feature (*p* < 0.001), and high FATP4 expression (*p* = 0.003) displayed an association with short recurrence-free survival (RFS, Figure 2(a)). In Kaplan-Meier survival analyses through the OncoLnc database, the high FATP4 expression was correlated with worse OS in RCC patients (*p* = 0.038, Figure 2(b)), while low CD36 and ACSL1 expressions were associated with poor OS (CD36, *p* = 0.038; ACSL1, *p* = 0.002).

In multivariate analyses, distant metastasis (hazard ratio = 10.16; 95% confidence interval, 1.68–61.42; *p* = 0.012) and tumor necrosis (hazard ratio = 7.49; 95% confidence interval, 1.97–28.57; *p* = 0.003) were independent factors for poor OS, while distant metastasis (hazard ratio = 12.63; 95% confidence interval, 3.41–40.86; *p* < 0.001), high grade (hazard ratio = 9.35; 95% confidence interval, 1.07–81.40; *p* = 0.043), high TNM stage (hazard ratio = 11.80; 95% confidence interval, 5.16–27.01; *p* < 0.001), and tumor necrosis (hazard ratio = 2.91; 95% confidence interval, 1.18–7.16; *p* = 0.02) were independent variables for reduced RFS. However, all three fatty acid transporters in this study reached no prognostic significance in multivariate survival analyses in terms of OS and RFS.

## 4. Discussion

This study showed that membranous expression of CD36 and FATP4 was higher in RCC tumor tissues than in normal tissues, whereas cytoplasmic ACSL1 was reduced in RCC tumor cells. These findings suggest that increased fatty acid transport across the cell membrane through CD36 and FATP4 might be a tumor-specific feature of RCC. Furthermore, the FATP4 expression was associated with poor prognostic factors of RCC such as high TNM stage, tumor necrosis, and shortened RFS, while the expressions of CD36 and ACSL1 were not associated. These results indicate that the high membranous expression of FATP4 in combination with CD36 might contribute to the tumorigenesis and tumor progression of RCC, and this tumor-specific alteration could be targeted for the treatment of RCC.

Deregulation of lipid metabolism is considered as one of the hallmarks of cancer. In addition to *de novo* fatty acid synthesis, cancer cells enhance exogenous fatty acid uptake to cope with increased demands for fatty acids in specific genetic and environmental conditions. For example, increased lipid uptake was observed in cells transformed by oncogenic *HRAS*, whereas cells transformed by constitutively active *AKT* increased *de novo* synthesis of fatty acids [19]. A report found that under conditions of metabolic stress such as hypoxia, extracellular lipid scavenging was an important adaptive mechanism for cancer cells to maintain viability and/or growth [20]. Another study showed that when cancer cells switch from a proliferative state to a migratory state, they lower *de novo* lipid biosynthesis and depend on exogenous lipid uptake [21]. Therefore, targeting *de novo* lipogenesis alone could give rise to treatment failure in some cancer patients.

The mammalian FATP family is composed of six members, FATP1 to FATP6 [22]. In various experimental models, FATPs have been suggested to promote the transport of long-chain fatty acids as a membrane transporter and/or as an enzyme with CoA synthetase activity [23, 24]. FATP4 is normally detectable in a wide variety of cells including heart, liver, brain, kidney, muscle, skin, and endothelial cells [25]. Within cells, FATP4 localized to the plasma membrane or other intracellular compartments such as the endoplasmic reticulum [26, 27]. In this study, FATP4 was weakly expressed in the plasma membrane and cytoplasm of normal tubular epithelial cells. Interestingly, membranous FATP4 was mostly found at the basal and apical membranes, which is beneficial for the transcellular migration of fatty acids considering the absorptive and secretory function of renal tubules.

Meanwhile, RCC tumor cells showed remarkable increases in membranous FATP4 and CD36 and a decrease in ACSL1, indicating that RCC cells are metabolically different from normal cells and prefer the mechanism of fatty acid uptake from extracellular sources during tumorigenesis. In addition, the high FATP4 expression was clearly associated with poor clinical outcomes of RCC patients in both our cohort and TCGA data, while low ACSL1 was correlated with poor OS in TCGA data. Consistent with this study, it was reported that HIF-2 downregulated the expression of both ACSL1 and carnitine palmitoyltransferase- (CPT-) 1 in rat hepatocytes, which induced the development of hepatic steatosis through the suppression of fatty acid oxidation in mitochondria [28]. Additionally, Du et al. reported that HIF-1 and HIF-2 repressed CPT1A in RCC cells and thereby diverted fatty acids from the mitochondria into cytoplasmic lipid droplets for storage [29]. Taken together, high membranous FATP4 and CD36 combined with low cytoplasmic ACSL1 in RCC tumor tissues may lower fatty acid utilization in the mitochondria and facilitate fatty acid uptake through the plasma membrane, resulting in cytoplasmic accumulation of lipid droplets and contributing to tumor progression.

In this study, increased membranous CD36 was not clinically significant in RCC, although a higher membranous expression of CD36 was revealed in tumor tissues than in normal cells. In contrast, the high CD36 expression was a factor predicting good OS in TCGA data analysis. The discrepancy regarding prognostic significance of CD36 between our cohort and TCGA data could be partly explained by the difference of detection targets and methods, because TCGA data included CD36 mRNA expression which was measured by RNA sequencing and had no information about the intracellular localization of CD36. Thus, it is possible that membranous CD36 indirectly assists with FATP4 in promoting fatty acid transport, leading to RCC progression. Similarly, other researchers have reported that the coexpression of CD36 with FATP4 led to a synergistic increase in oleate uptake in Madin-Darby canine kidney cells [14]. However, additional research is required to determine whether CD36 carries an independent prognostic significance in RCC and FATP4 and CD36 colocalize at the same area of the cell membrane and form a protein complex.

In conclusion, this study showed for the first time that the high FATP4 expression is associated with poor prognostic factors of RCC. Increased fatty acid uptake based on the high membranous expression of FATP4 and CD36 can be specifically targeted in RCC tumor cells in combination with other therapies against altered lipid metabolism. The precise molecular mechanism of fatty acid transport in human malignancies including RCC should be investigated further.

## Figures and Tables

**Table 1:** CD36, FATP4, and ACSL1 expressions in normal and RCC tumor tissues.

	Normal (mean ± SD) (*n* = 80)	RCC (mean ± SD) (*n* = 180)	*p* value
CD36 (IM score)	6.36 ± 1.58	13.14 ± 3.62	<0.001
FATP4 (IM score)	7.84 ± 2.10	10.28 ± 4.00	<0.001
ACSL1 (percentile of stained area)	29.32 ± 13.57	1.23 ± 3.97	<0.001

RCC: clear cell renal cell carcinoma; SD: standard deviation; IM: immunomembrane.

**Table 2:** CD36, FATP4, and ACSL1 expressions in RCC cases.

Characteristics	CD36 (%)			FATP4 (%)			ACSL1 (%)		
	High (*n* = 74)	Low (*n* = 106)	*p* value	High (*n* = 85)	Low (*n* = 95)	*p* value	High (*n* = 72)	Low (*n* = 108)	*p* value
Age									
Low (<60 yr)	42 (56.8)	60 (56.6)	0.984	43 (50.6)	59 (62.1)	0.120	40 (55.6)	62 (57.4)	0.806
High (≥60 yr)	32 (43.2)	46 (43.4)		42 (49.4)	36 (37.9)		32 (44.4)	46 (42.6)	
Gender									
Male	52 (70.3)	75 (70.8)	0.944	54 (63.5)	73 (76.8)	0.05	44 (61.1)	83 (76.9)	0.023
Female	22 (29.7)	31 (29.2)		31 (36.5)	22 (23.2)		28 (38.9)	25 (23.1)	
Grade									
Low	12 (16.2)	22 (20.8)	0.444	13 (15.3)	21 (22.1)	0.244	12 (16.7)	22 (20.4)	0.534
High	62 (83.8)	84 (79.2)		72 (84.7)	74 (77.9)		60 (83.3)	86 (79.6)	
TNM stage									
Low (I, II)	58 (78.4)	87 (82.1)	0.537	63 (74.1)	82 (86.3)	0.039	55 (76.4)	90 (83.3)	0.249
High (III, IV)	16 (21.6)	19 (17.9)		22 (25.9)	13 (13.7)		17 (23.6)	18 (16.7)	
Metastasis									
Absent	71 (95.9)	99 (93.4)	0.529	80 (94.1)	90 (94.7)	1.000	67 (93.1)	103 (95.4)	0.524
Present	3 (4.1)	7 (6.6)		5 (5.9)	5 (5.3)		5 (6.9)	5 (4.6)	
Tumor necrosis									
Absent	65 (87.8)	95 (89.6)	0.708	70 (82.4)	90 (94.7)	0.009	64 (88.9)	96 (88.9)	1.000
Present	9 (12.2)	11 (10.4)		15 (17.6)	5 (5.3)		8 (11.1)	12 (11.1)	
Sarcomatoid feature									
Absent	70 (94.6)	101 (95.3)	1.000	81 (95.3)	90 (94.7)	1.000	70 (97.2)	101 (93.5)	0.319
Present	4 (5.4)	5 (4.7)		4 (4.7)	5 (5.3)		2 (2.8)	7 (6.5)	
Recurrence									
Absent	61 (82.4)	86 (81.1)	0.824	64 (75.3)	83 (87.4)	0.037	61 (84.7)	86 (79.6)	0.387
Present	13 (17.6)	20 (18.9)		21 (24.7)	12 (12.6)		11 (15.3)	22 (20.4)	

RCC: clear cell renal cell carcinoma; TNM: tumor-node-metastasis.

## Data Availability

The data used to support the findings of this study are available from the corresponding author upon request.
